# Molecules promoting circulating clusters of cancer cells suggest novel therapeutic targets for treatment of metastatic cancers

**DOI:** 10.3389/fimmu.2023.1099921

**Published:** 2023-03-15

**Authors:** Julian M. Rozenberg, Anton A. Buzdin, Tharaa Mohammad, Olga A. Rakitina, Dmitry A. Didych, Victor V. Pleshkan, Irina V. Alekseenko

**Affiliations:** ^1^ Laboratory of Translational Bioinformatics, Moscow Institute of Physics and Technology, Dolgoprudny, Russia; ^2^ PathoBiology Group, European Organization for Research and Treatment of Cancer (EORTC), Brussels, Belgium; ^3^ Group for Genomic Analysis of Cell Signaling, Shemyakin-Ovchinnikov Institute of Bioorganic Chemistry of the Russian Academy of Sciences, Moscow, Russia; ^4^ Laboratory for Clinical Genomic Bioinformatics, Sechenov First Moscow State Medical University, Moscow, Russia; ^5^ Gene Immunooncotherapy Group, Shemyakin-Ovchinnikov Institute of Bioorganic Chemistry of the Russian Academy of Sciences, Moscow, Russia; ^6^ Laboratory of human genes structure and functions, Shemyakin-Ovchinnikov Institute of Bioorganic Chemistry of the Russian Academy of Sciences, Moscow, Russia; ^7^ Gene oncotherapy sector, Institute of Molecular Genetics of National Research Centre (Kurchatov Institute), Moscow, Russia; ^8^ Laboratory of Epigenetics, Institute of Oncogynecology and Mammology, National Medical Research Center for Obstetrics, Gynecology and Perinatology Named after Academician V.I. Kulakov, Ministry of Healthcare of the Russian Federation, Moscow, Russia

**Keywords:** cancer, metastasis, circulating cancer cell, tumor microenvironment, heterotypic cell interactions

## Abstract

Treatment of metastatic disease remains among the most challenging tasks in oncology. One of the early events that predicts a poor prognosis and precedes the development of metastasis is the occurrence of clusters of cancer cells in the blood flow. Moreover, the presence of heterogeneous clusters of cancerous and noncancerous cells in the circulation is even more dangerous. Review of pathological mechanisms and biological molecules directly involved in the formation and pathogenesis of the heterotypic circulating tumor cell (CTC) clusters revealed their common properties, which include increased adhesiveness, combined epithelial-mesenchymal phenotype, CTC-white blood cell interaction, and polyploidy. Several molecules involved in the heterotypic CTC interactions and their metastatic properties, including IL6R, CXCR4 and EPCAM, are targets of approved or experimental anticancer drugs. Accordingly, analysis of patient survival data from the published literature and public datasets revealed that the expression of several molecules affecting the formation of CTC clusters predicts patient survival in multiple cancer types. Thus, targeting of molecules involved in CTC heterotypic interactions might be a valuable strategy for the treatment of metastatic cancers.

## 1 Introduction

One of the cancer hallmarks is cancer cells dissemination and metastasis which is a leading cause of cancer associated death (1). Metastasis develops as a consequence of changes within cancer cells that lead to an ability to move through the tissue, survive in the circulation, attach and grow in the distal site, meanwhile escaping immune surveillance (2). Research of the last decades revealed that a key factor which determines the ability of cancer cells to metastasize is pathological interactions with neighboring non-cancerous cells such as fibroblasts, mesenchymal and immune cells, so called cells of tumor microenvironment (TME).

Therefore, development of drugs targeting key molecules involved in the TME interactions that can suppress metastasis is a hot theme of current investigations (3–7).

Cancer associated stromal cells as well as circulating exosomes migrate from the primary tumor to distal sites and change local microenvironment forming so-called pre-metastatic niche permissive for the cancer cells recruitment and growth (8–12). At the same time, cancer cells might disseminate from the primary tumor in the circulation in clusters with cancer associated cells (13–15). These clusters are thought to be relatively rare in the cancer patient population (14, 16, 17), although they have strong metastatic potential (16, 18), and their presence is associated with metastasis and worse prognosis in breast (13, 14, 19), lung cancers (20–22), renal cell carcinoma (23), colorectal cancer (24–26), and others (27).

Our review of current literature revealed that cells involved in the metastasis-promoting heterotypic CTC interactions include platelets, cancer associated fibroblast (CAFs), white blood cells (WBCs), specific population of tumor-associated macrophages, neutrophils and polymorphonuclear myeloid-derived suppressor cells (PMN-MDSCs).

A number of investigations identified several key molecules involved in the heterotypic cell interactions such as IL1R1 (16), IL6, NODA, NOTCH1 (17), CD44 (14), CXCR4 (4), TGFBR2 (4), CDH1 (4, 28), EPCAM (29), ICAM1 (30), CCR1 (31) (**Table 1**). Their expression promotes formation of CTC clusters and metastasis by inducing adhesion (4, 30, 33), proliferation (16), by metabolic adaptation to oxidative stress (17, 34), and through the epithelial-mesenchymal transition (31, 35). Quite intriguing, in lung cancer most CTCs interacting with WBCs were polyploid (21) thus, implying repression of the mitotic checkpoint, induction of cell survival and migration (36–38).

**Table 1:** Genes involved in cancer cell-stromal cell interaction promoting CTC clustering and metastasis.

Cancer type	Target	Target ligand(s)	Interacting cells	Source tissue	Reference
Breast	IL6ST	IL6	neutrophils	Peripheral blood	(16)
Breast	IL1R1	IL1	neutrophils	Peripheral blood	(16)
Breast	VCAM1	ITGA4 ITGB1	neutrophils	Peripheral blood	(16)
Breast	NODAL	CFC1B	PMN-MDSCs	Spheroid cell co-culture	(17)
Breast	NOTCH1	JAG1	PMN-MDSCs	Spheroid cell co-culture	(17)
Breast	CD44	Hyaluronic acid	CAFs	MDA-MB-231 and CD44 positive MCF-7 cells	(14)
Breast	CXCR4	CXCL12	CAFs	MCF10DCIS	(4, 32)
Breast	TGFBR2	TGFB1	CAFs	MCF10DCIS	(4)
Hepatocellular	EPCAM	CAMs	NA	Huh7 organoids in xenograft model	(29)
Lung	ICAM1	ITGAM	PMNs and neutrophils	Lewis lung carcinoma H-59 cells, A549 cells expressing ICAM-1	(30)
Colorectal	IL6R	IL6	Tumor-associated macrophages (TAMs)	Patient blood	(31)
Colorectal	CCR1	CCL2	Tumor-associated macrophages (TAMs)	Patient blood	(31)
Colorectal	CDH1	CDH1, adherent junction protein	NA	Human CRC organoids in xenograft model	(28)

The analysis of literature and public databases revealed that expression of some genes affecting CTC clusters and metastasis predicts prognosis in many cancer types. Some of these molecules are targeted by the approved or experimental anti-cancer drugs (such as plerixafor for CXCR4 or tocilizumab for IL6R). Altogether, our review suggests the existence of common and cancer tissue specific mechanisms of CTC complex formation with implication for drug development and cancer treatment.

## 2 Tumor microenvironment promotes formation of the CTC clusters and metastasis

### 2.1 Interactions with white blood cells

The CTCs can interact with a variety of WBCs in the circulation such as neutrophils (39), PMN-MDSC (17, 40, 41), platelets (31), macrophages (35), and lymphocytes (16).

#### 2.1.1 Interaction with neutrophils

One of the mechanisms of neutrophil mediated metastasis is formation of the neutrophil extracellular traps (NETs) consisting of neutrophil DNA (39). As NETs interact with and provide a niche for CTCs, blocking NET formation by DNAse, e.g. coated with nanoparticles inhibits lung metastasis (39).

Using *in vivo* metastasis models, Spicer et al. have demonstrated a novel role of neutrophils in the early adhesive steps of liver metastasis in the Lewis lung carcinoma mice model (30). Their findings suggest that neutrophils promote cancer cell adhesion within liver sinusoids, thus influencing metastasis. The neutrophil ITGAM/ICAM-1 mediated the adhesion of lipopolysaccharide-activated neutrophils to the cancer cells (30).

In breast cancer, CTCs interact with WBCs and in out of 70 investigated patients with invasive disease, CTCs were found in 34 (49%) patients. Among them, homotypic CTC clusters were found in 14 (20%) patients, out of which 6 (9%) also had CTC-WBC clusters and 4 (6%) had CTC-WBC clusters only (16). On average, about 2 CTCs were found in the CTC-WBC clusters that represented about 10% of all circulating CTCs (16). Most of these WBCs (75%) were myeloid cells, specifically neutrophils and T-cells.

The neutrophil-CTC interactions detected in blood were associated with worse prognosis of patients (16). Neutrophil-CTC clusters promoted cancer cell proliferation *in vitro* and were characterized by higher metastatic potential in mice upon tail vein injection. Analysis of gene expression from either CTC alone or in a complex with neutrophils revealed 41 upregulated genes involved in the DNA replication and cell cycle progression. Further analysis of genes dysregulated in cancer associated neutrophils revealed that TNF-α, Oncostatin M (OSM), IL-1β and IL-6 cytokines are expressed in the neutrophils with corresponding expression of their receptors in CTCs. Reciprocal experiment detected cytokines granulocyte colony-stimulating factor (G-CSF), TGF-β3 and IL-15 in the CTCs with corresponding expression of the receptors in neutrophils. CRISPR-Cas9 mediated knockout of IL6ST and IL1R1 in cancer cells suppressed the growth advantage of the neutrophil-CTC clusters without effect on their frequency (16). In addition, vascular cell adhesion molecule (VCAM1) was identified in a CRISPR-Cas9 screen in the CTC as a molecule required for formation of the neutrophil-CTC clusters (16). Neutrophil recruitment to the primary site and metastasis was dependent on expression of CXCL1/2 in 4T1 breast cancer cells. Among molecules that block cancer cell invasion mediated by neutrophils were also NADPH oxidase, neutrophil elastase inhibitors, and DNAse (39).

#### 2.1.2 Interaction with PMN-MDSCs

Another type of myeloid cell - PMN-MDSCs normally function as suppressors of the immune response and have profound pro-carcinogenic properties promoting angiogenesis, formation of the pre-metastatic niche and cell proliferation (42–45),

It was predicted that PMN-MDSCs interact with CTCs and it was hypothesized (yet to be proven) that PMN-MDSCs shield CTCs from the T-cell mediated destruction (46). At that time, CTCs were usually isolated as CD45 negative cells, thereby clusters of CTC with leukocytes (including PMN-MDSCs) were missed from the analysis.

Indeed, PMN-MDSC clusters with circulating tumor cells were detected in patients with melanoma or breast cancer (17). It was reported that the ratio of cancer and non-cancerous cells in the clusters varied in the range 1:1 to 1:4 in six out of eight patients tested (17).

Interestingly, a previous paper from the same group revealed that aggressive triple negative breast and melanoma cancers overexpress Nodal, an embryonic morphogen of the TGF-β family (47) and a a putative Notch/RBPJ signaling pathway target (48). The patients with aggressive breast cancer had higher levels of Nodal in serum and PMN-MDSCs could promote survival of the CTCs in culture by activating reactive oxygen species (ROS) and Jagged2 response (17). Accordingly, CTCs promote differentiation of the PMN-MDSCs in pro-cancerous “type-2” phenotype by the Nodal signaling (17).

Arnoletti et al. investigated the effect of interactions between the CTCs, MDSCs and T-cells extracted from the portal blood of pancreatic adenocarcinoma patients on CTC and T-cell proliferation, apoptosis and activation. It was demonstrated that MDSCs tended to cooperate with CTCs by repressing T-cells proliferation, although no significant effects on activation and anergy were reported (49).

The mathematical modeling and direct measurements of genomic aberrations in breast cancer CTC clusters isolated by filtration revealed that the fraction of cancer cells in the clusters is in the range of 8%-48% (50). In contrast, isolation of multicellular clusters from the blood of breast cancer patients followed by single cell RNA-seq analysis identified genes associated specifically with clusters, in comparison to single cells, but failed to identify other cell types except platelets (18). In agreement with other studies, cell clusters contributed to metastasis 23 times more actively than the single cells and the presence of clusters in breast and prostate cancers was associated with significantly worse prognosis (18).

The differences in CTC isolation protocols might lead to the differences in cell populations detected within CTC clusters. The latter study (18) utilized HBCTC-Chip coated with cocktail of EPCAM, EGFR and HER2 antibodies (18), whereas Parsortix microfluidic device using Cell Separation Cassettes (GEN3D6.5, ANGLE) was used in the subsequent study that characterized neutrophils-CTC (16), whereas PMN-MDSC-CTCs clusters were isolated by FACS (17, 40, 41).

#### 2.1.3 Interaction with tumor associated macrophages

Interaction of CTCs with tumor associated macrophage (TAMs) seems to promote metastasis. Nanomechanical characterization of tumor associated macrophage-CTC clusters isolated from blood of prostate cancer patients revealed that contact with the macrophages softens and promotes adhesiveness of CTCs, which corresponds to mixed epithelial - mesenchymal phenotype (35). Notably, previous publication of the same group reported softness, deformability, and adhesiveness of single CTCs as markers of aggressive metastatic prostate cancer (51). The presence of TAMs in the invasive front was associated with the mesenchymal phenotype of CTCs and poor prognosis in colorectal cancer (31). Mechanistically, the Il-6 produced by the TAMs induced JAK2/STAT3/miR-506-3p/FoxQ1 signaling in cancer cells, thus promoting epithelial mesenchymal transition (EMT), metastasis and further attraction of macrophages by secretion of CCl2 (31).

#### 2.1.4 Interaction with lymphocytes

We found only one report that mentions interaction of CTCs with lymphocytes (16). However, CTCs are associated with impairments of adaptive immunity. The quantity of CTCs correlates with the presence in peripheral blood of the CD95(FAS)-positive T-helper cells and stage 3 breast cancer as well as with lower percentage of the CD8+ T-cells with activated T-cell receptor (52, 53), the absence of tumor associated antigen specific TCRs and low TCR heterogeneity (54), and positively associated with intratumoral populations of T-regs (55).

### 2.2 Interactions with cancer associated fibroblasts

Aside from single CTCs and cancer associated fibroblasts (CAFs), the presence of homotypic and heterotypic clusters of CTCs and CAFs was reported in patients with stages 1-4 of breast cancer (14). In their study, Sharma et al. detected CTCs in 90% and circulating CAFs (cCAFs) in 80% of patients; homotypic CTC clusters were found in 50% and heterotypic - in 25% of patients in treatment naive stages 2-3. Interestingly, only 25% of patients in stage 4 had homotypic clusters and 25% had heterotypic CTC-CAF clusters. The number of cCAFs and CTCs was much higher in patient blood with metastatic breast cancer in comparison to localized cancers whereas nothing was detected in the control group. The effect of cancer treatment on these clusters was not yet addressed (14).

Using MDA-MB-231 cells and CD44-enriched MCF7 cells, authors have been able to demonstrate involvement of the stem cell marker CD44 in the heterotypic clustering and that heterotypic clusters metastasize more efficiently (14). Accordingly, it was shown that tumor suppressor Rb represses CD44 dependent collective invasion, release of breast cancer cells in circulation and lung metastasis (3).

Circulating CAFs and CTCs were also detected in small groups of colorectal and prostate cancer patients (13). Consistent with others, the paper shows images of the distinct multicellular CTC clusters with CAF and with leukocytes, which were obtained by the negative filtration through 10 µm filter (13).

### 2.3 Interaction with platelets

Activation of the coagulation cascade and formation of platelet-rich thrombus around tumor cells in the vasculature have both been proposed to play major roles in physically shielding CTCs from the stress of blood flow and from lysis by the Natural killer cells (56–58). One of the mechanisms is substitution of cancer cell MHC1 by platelets-derived MHC1 carrying normal peptides thereby protecting cancer cells from both NK and T-cell recognition (59).

Analysis of the single cell gene expression of the CTCs in the pancreatic cancer mouse model revealed that 32% of the circulating cells interact with platelets leading to suppression of epithelial markers and expression changes of many other genes (60).

Accordingly, direct interaction with platelets promotes EMT in cancer cells and either inhibition of NF-kB in cancer cells or inhibition of TGF-β in platelets was sufficient to protect against lung metastasis (61). In turn, disruption of platelets interactions with cancer cell by S-nitrosocaptopril (CapNO) inhibits adhesion to endothelial cells and lung cancer metastasis in immunocompetent mouse models through multiple mechanisms including reduction of Sialyl-Lewis X (Slex) levels in cancer cells and ADP-induced P-selectin in platelets, IL-1b induced VCAM1, ICAM-1, and E-selectin by HUVECs (33).

## 3 Polyploidy and epithelial-to-mesenchymal transition in CTC clusters

As it is discussed in the previous sections, interaction with TAM (31) or platelets (61) induced metastasis promoting EMT in cancer cells (62). EMT is associated with cancer progression and metastasis (63). During EMT epithelial cells lose contact with epithelial or endothelial cells, change their cytoskeleton and consequentially, become less rigid, acquiring an ability to move (51, 64). In addition, EMT induces stem cell properties in cancer, regulates and is regulated by immunosuppressive cancer microenvironment (65, 66). Notably, cancer stem cells are characterized by mixed epithelial – mesenchymal phenotype (67).

Interestingly, interaction with white blood cells also correlates with mixed Epithelial-mesenchymal phenotype and cancer cells polyploidy (21, 68, 69) that play a key role in cancer resistance to treatment and metastasis (37, 70–72).

The presence of CTC-WBC clusters was associated with worse prognosis in lung (21, 22), breast cancers (19), and hepatocellular carcinoma (73, 74). Remarkably, in lung cancer, CTCs in complex with WBCs were exclusively polyploid (21).

In turn, in glioblastoma, examination of ploidy together with expression of endothelial marker CD31 revealed that pre-operative small triploid CD31 negative CTCs were predictive of inferior prognosis (68).

A recent paper employed the iFISH method combining FISH DNA staining and immunofluorescence (21, 22) to create Atlas of Circulating Rare Cells (69). High throughput imaging analysis of circulating rare cells (CRCs) purified by WBC subtraction categorized cells into 71 subtypes based on the CD45 leukocyte staining, cell size, chromosome 8 ploidy and the presence of a few tumor cell markers including PD-L1 (EPCAM/CK18/PD-L1/AFP/HER2/CA19-9), endothelial CD31, mesenchymal Vimentin and stem cell CD133 markers (69).

Authors presented a set of cell images with polyploid chromosome 8. There were cells double positive for CD31 and Vimentin staining with abnormal chromosomes which can coincide with cytokeratin CK18, and even CD45-/EPCAM+/CD31+/Vim+ “aneuploid mesenchymal epithelial-endothelial fusion clusters” were detected. These observations are consistent with the previous data generated by iFISH linking polyploidy with EMT (21, 75, 76). The presence of CD45 positive cells was detected in the clusters with polyploid or multinuclear cancer cells (21, 69).

Quite importantly, comparison of the total count of CTCs and/or circulating tumor endothelial cells between 31 conditions revealed that CTCs are present in multiple cancers, however, the highest frequency of “CTCs” is observed within the group of non-neoplastic infectious diseases, suggesting that the pure presence of cells with these markers could not be used as a diagnostic test itself (69).

Consistent with the Atlas of Circulating Rare Cells (69), sequencing of CTC clusters and individual circulating cancer cells revealed the mixed epithelial-mesenchymal markers in hepatocellular carcinoma (Vimentin, epithelial: CDH1, EPCAM, ASGR2, Keratin 8, stemness: CD133, POU5F1, NOTCH1 and STAT3) (62) prostate cancer (EPCAM, keratins, E-cad, Vimentin, CD44) (77) and Vimentin in lung cancer (78).

However, two major conceptual questions here currently remain not sufficiently addressed:

(i) How heterotypic interactions of cancer cells with WBCs promote polyploidy?
(ii) How does the combination of ploidy and mesenchymal phenotype enhance metastasis?

Mechanisms of how heterotypic interactions promote mobility and mesenchymal phenotype are described in the subsequent section.

## 4 Heterotypic interactions within tumor microenvironment are pivotal for CTC cluster formation

Interactions with cells of cancer microenvironment promote EMT, formation of CTC clusters and metastasis (4, 31). Classically, EMT is accompanied by decrease of E-cadherin/N-cadherin ratio (79). A recent publication highlighted a novel role of the E-cadherin (E-cad, encoded by CDH1 gene) expressing cells in breast cancer metastasis (80, 81). It turned out that when cancer cells grow in the presence of CAFs there is a gradient of the E-cad from low at the trailing edge of the invading cancer cells to high E-cad behind it (4). Furthermore, another paper demonstrated that in breast cancer spheroid model stem cells lead the collective invasion co-expressing mesenchymal and epithelial marks (82).

Dermal implants of CAFs with MCF10 cells with low intrinsic metastatic potential promoted this low-high E-cad gradient, the CTC cell clustering and metastasis (4). High throughput RNA expression profiles revealed induction of carcinoembryonic antigen-related cell adhesion molecule 5 (CEACAM5; CAM5) and CEACAM6 (CAM6) in the presence of CAFs. This experiment revealed overexpression of 44 CAF-induced genes, whose expression is associated with poor prognosis in breast cancer. Mechanistically, E-cad, CAM5 and CAM6 interact with each other forming an adherent junction complex on the cell surface. Functional shRNA studies revealed attenuation of lung metastasis upon E-cad, CAM5, or CAM6 depletion. Other excellent functional investigations reported in this paper revealed that CAF produced SDF-1(encoded by CXCL12 gene) and TGF-β that through their cognate receptors CXCR4 and TGFBRII activate SRC kinase phosphorylation/Zeb1 axis altogether mediating tumor cell cluster formation that are also detected as CTC clusters. The caveat of this report for our purposes is that we do not know if fibroblasts travel in the bloodstream with cancer cells. However, this paper clearly demonstrates stromal-cancer cell molecular interactions that regulate the ability of cancer to metastasize (4). Importantly, CRCX4 mediates immunosuppressive tumor microenvironment not only in cancer cells, but also in the SMA positive stromal cells including myofibroblasts and pericytes (32). CRE-Lox mediated knockout of CRCX4 in SMA expressing cells improved survival in mice with breast cancer, and pharmacological inhibition of CRCX4 potentiated activity of immune checkpoint inhibitors in the nude mice bearing human metastatic breast cancer (32).

Similarly, to observation in breast cancer, cells of the collective invasion packs were E-cad positive in lung adenocarcinoma (5). The role of CAFs in the metastasis was demonstrated by the fact that only surrounding CAFs express vimentin and in the vimentin knockout mice, the CAFs motility decreases *in vitro* and *in vivo*. Vimentin was required for the heterotypic cancer cell - CAFs interaction, collective invasion, and lung adenocarcinoma metastasis (5).

Thus, formation of Epithelial-mesenchymal gradient during collective invasion is mediated by cancer cell – stromal cell interaction and pivotal for CTC formation and metastasis (4, 5).

**Figure 1:**
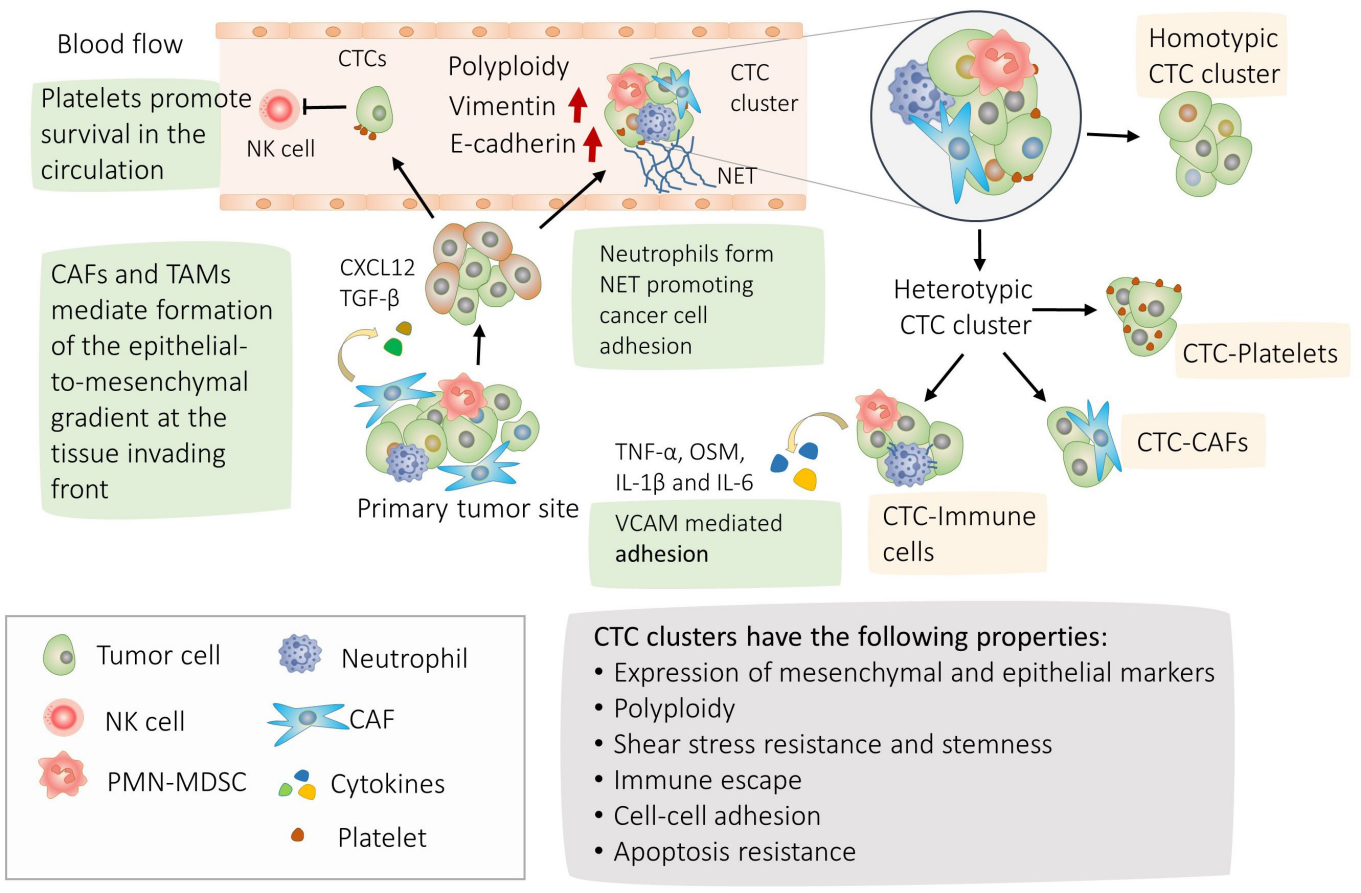
Mechanisms of circulating tumor cells (CTC) cluster formation and their properties. Highlighted in green, the process of CTC clusters formation; Tumor cells can separate from adjacent cells with more mobile mesenchymal cells at the leading edge of the invasion and cells with more epithelial properties behind (4, 5). Accordingly, mixed epithelial mesenchymal phenotype and polyploidy are frequently observed in the CTC clusters (21, 22). CTCs and CTC clusters are able to withstand the shear stress in the blood circulation and escape natural killer (NK). CTCs can form homotypic clusters or interact with CAFs, neutrophils, PMN-MDSCs, Tumor associated macrophages (TAM) or platelets forming heterotypic clusters (14, 16, 17). CAFs circulate in the bloodstream in heterotypic CTC clusters and promote cancer cell clustering by secreting CXCL12 and TGF-β (4, 13, 20). During transit and metastasis, platelet-rich thrombus form around CTCs providing protection from shear stress and against lysis by NK cells (56–58). CTC-associated neutrophils express TNF-α, OSM, IL-1β and IL-6 cytokines and their receptors are expressed correspondingly in CTCs. The interaction between CTCs and neutrophils is mediated by VCAM1, whereas TNF-α, OSM, IL-1β, and IL-6 promote proliferation of CTCs (16). In addition, neutrophils promote metastasis by releasing their DNA forming neutrophil extracellular traps (NET) (39). In turn, interaction with polymorphonuclear myeloid derived suppressor cells promote survival of the CTC clusters (17).

**Figure 2:**
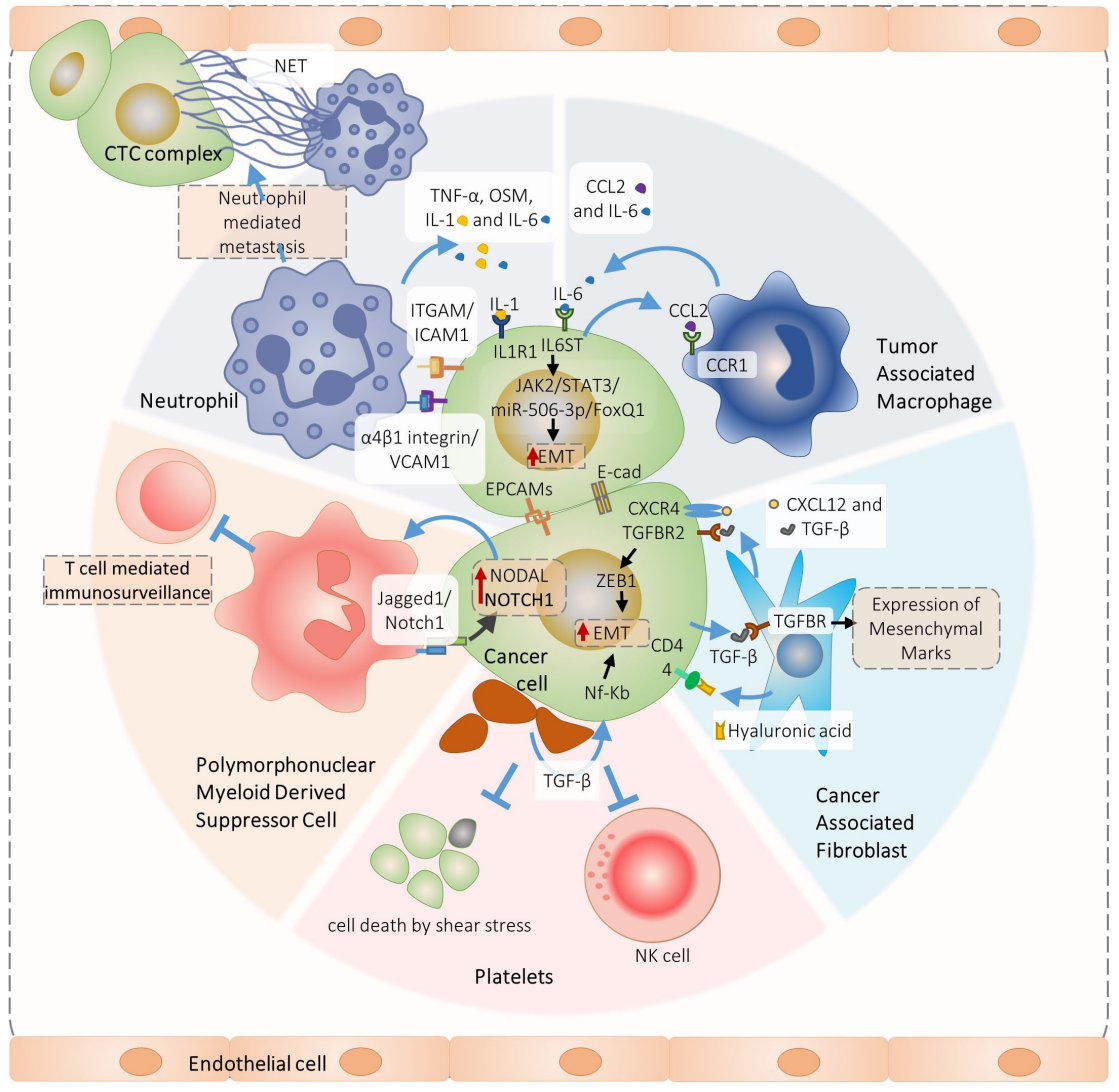
Overview of signaling pathways involved in the heterotypic cancer cell interactions pivotal for circulating tumor cells complex formation and metastasis. Specifically, PMN-MDSC-cancer cell interaction promotes ROS-induces Jagged1/Notch1/Nodal signaling that induces CTC cluster formation and metastasis (17), In turn, neutrophil interact with cancer cells *via* ITGAM/ICAM-1 adhesion facilitating interaction with liver sinusoids and metastasis (30). In addition, VCAM1 is required for the neutrophil-CTC cluster formation (29). Neutrophils produce IL1 and IL-6 that promote growth of neutrophils-CTC clusters *via* IL6ST and IL1R1 receptors (16), The Il-6 is also produced by the tumor associated macrophages which induce JAK2/STAT3/miR-506-3p/FoxQ1 signaling in cancer cells promoting epithelial mesenchymal transition (EMT), metastasis and further attraction of macrophages by the CCl2 secretion (31). A similar positive feedback loop is organized by the cancer-associated fibroblasts (CAFs) and cancer cells interactions. The CAFs produce TGF-β and CXCL12 that interact with TGFBR2 and CXCR4 receptors, inducing cancer cell EMT, CTC clusters and metastasis (4). In turn, cancer cells produce TGF-β and induce CAFs myofibroblast differentiation (83).

We schematized major findings on CTC interactions and their molecular physiological effects on **Figures 1**, **2**.

## 5 Expression of molecules involved in the CTC cluster formation and metastasis correlate with cancer survival

**Figure 3:**
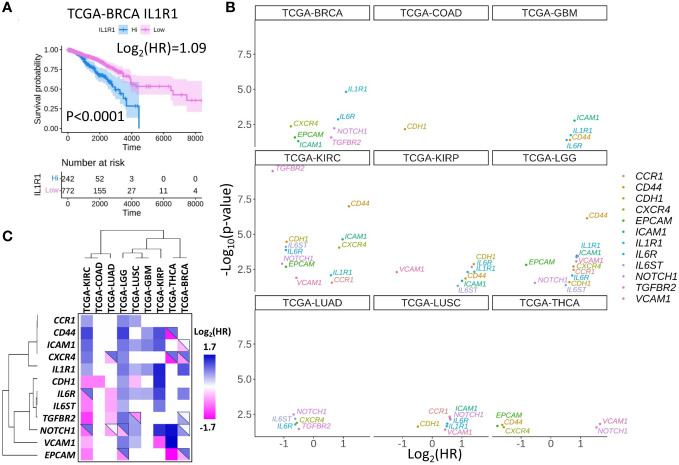
Set of selected CTC-associated genes differentially predict survival in several human cancer types. **(A)** An example of Kaplan-Meier curve for breast cancer (BRCA) patients stratified by high or low *IL1R1* expression in tumors. Shaded areas represent 95% confidence intervals. Time is shown in days. **(B)** Hazard ratios (HR) and significance of the differences between Kaplan-Meier curves for patients stratified by the expression of indicated genes for the panel of solid tumors in TCGA database. Only genes with p<0.05 are shown. Note that log_2_(HR)<0 for good prognosis corresponding to high gene expression and log_2_(HR)>0 for bad prognosis. **(C)** Hazard ratio clustering for gene - cancer combinations revealed two major gene clusters. Top-right triangles depict approximations of the HR collected from the literature. Blue colors represent genes or gene products whose expression is associated with worse prognosis, pink colors represent genes or gene products whose expression is associated with good prognosis while white colors represent cases where the data is controversial.

As it is discussed in the previous sections, the formation of the CTC clusters and metastasis in particular cancers depend on IL1R1 (16), IL6, NODAL, NOTCH1 (17), CD44 (14), CXCR4 (4), TGFBR2 (4), CDH1 (4, 28), EPCAM (29), ICAM1 (30), and CCR1 (31). Theoretically, these molecules can impact cancer metastasis with little to no information on the mechanisms involved in CTC cluster formation. To address this possibility, we interrogated a publicly available The Cancer Genome Atlas project (TCGA) database and research papers to examine if high or low expression of molecules that are functionally important for the formation of CTC clusters may characterize patient survival in multiple cancers. For example, it was demonstrated that IL1R1 protein induces CTC proliferation in breast cancer (BRCA) (16), and high *IL1R* gene expression corresponds to inferior prognosis in the TCGA-BRCA cohort (**Figure 3A**) as well as in many other cancers (**Figures 3B, C**; **Table 2**). In turn, high expression of *CXCR4* in BRCA corresponds to better prognosis, smaller yet significant difference between Kaplan-Meier curves predicting better prognosis was observed for lung adenocarcinoma (LUAD) and thyroid cancer (THCA) (**Figure 3B**, **Table 2**), whereas no difference was observed in lung squamous cell carcinoma (LUSC).

**Table 2:** *p*-values and Hazard ratio (HR) levels for survival of patients with high gene expression in tumors depicted in **Figures 3B, C**.

Gene name	TCGA project ID	p-value	HR	log_2_(HR)
CCR1	TCGA-KIRC	0.0283	0.65	-0.61
CCR1	TCGA-LGG	0.0035	0.59	-0.77
CCR1	TCGA-LUSC	0.0047	0.67	-0.59
CD44	TCGA-GBM	0.0408	0.64	-0.64
CD44	TCGA-KIRC	0.0000	0.44	-1.18
CD44	TCGA-KIRP	0.0143	0.46	-1.12
CD44	TCGA-LGG	0.0000	0.43	-1.22
CD44	TCGA-THCA	0.0181	3.10	1.63
CDH1	TCGA-COAD	0.0070	1.89	0.92
CDH1	TCGA-KIRC	0.0000	1.88	0.91
CDH1	TCGA-KIRP	0.0014	0.38	-1.40
CDH1	TCGA-LGG	0.0255	0.65	-0.63
CDH1	TCGA-LUSC	0.0240	1.40	0.48
CXCR4	TCGA-BRCA	0.0044	1.70	0.76
CXCR4	TCGA-KIRC	0.0001	0.55	-0.86
CXCR4	TCGA-LGG	0.0020	0.59	-0.76
CXCR4	TCGA-LUAD	0.0129	1.47	0.56
CXCR4	TCGA-THCA	0.0266	2.99	1.58
EPCAM	TCGA-BRCA	0.0269	1.54	0.63
EPCAM	TCGA-KIRC	0.0021	1.90	0.93
EPCAM	TCGA-LGG	0.0015	1.77	0.83
EPCAM	TCGA-THCA	0.0217	z3.47	1.80
ICAM1	TCGA-BRCA	0.0493	1.43	0.52
ICAM1	TCGA-GBM	0.0017	0.58	-0.80
ICAM1	TCGA-KIRC	0.0000	0.51	-0.97
ICAM1	TCGA-KIRP	0.0214	0.48	-1.06
ICAM1	TCGA-LGG	0.0003	0.55	-0.87
ICAM1	TCGA-LUSC	0.0066	0.66	-0.60
IL1R1	TCGA-BRCA	0.0000	0.47	-1.09
IL1R1	TCGA-GBM	0.0186	0.63	-0.67
IL1R1	TCGA-KIRC	0.0084	0.67	-0.58
IL1R1	TCGA-KIRP	0.0047	0.38	-1.41
IL1R1	TCGA-LGG	0.0004	0.55	-0.86
IL1R1	TCGA-LUSC	0.0226	0.71	-0.49
IL6R	TCGA-BRCA	0.0014	0.57	-0.82
IL6R	TCGA-GBM	0.0415	0.69	-0.53
IL6R	TCGA-KIRC	0.0001	1.91	0.93
IL6R	TCGA-KIRP	0.0050	0.44	-1.19
IL6R	TCGA-LGG	0.0088	0.61	-0.72
IL6R	TCGA-LUAD	0.0166	1.53	0.62
IL6R	TCGA-LUSC	0.0148	0.70	-0.50
IL6ST	TCGA-KIRC	0.0001	1.91	0.93
IL6ST	TCGA-KIRP	0.0474	0.55	-0.86
IL6ST	TCGA-LGG	0.0422	0.71	-0.50
IL6ST	TCGA-LUAD	0.0067	1.54	0.62
NOTCH1	TCGA-BRCA	0.0060	0.62	-0.69
NOTCH1	TCGA-KIRC	0.0013	2.08	1.06
NOTCH1	TCGA-KIRP	0.0021	0.40	-1.33
NOTCH1	TCGA-LGG	0.0292	1.45	0.54
NOTCH1	TCGA-LUAD	0.0033	1.59	0.67
NOTCH1	TCGA-LUSC	0.0074	0.65	-0.62
NOTCH1	TCGA-THCA	0.0275	0.35	-1.53
TGFBR2	TCGA-BRCA	0.0271	0.66	-0.59
TGFBR2	TCGA-KIRC	0.0000	2.60	1.38
TGFBR2	TCGA-LGG	0.0004	0.53	-0.91
TGFBR2	TCGA-LUAD	0.0350	1.41	0.50
TGFBR2	TCGA-LUSC	0.0050	0.67	-0.58
VCAM1	TCGA-KIRC	0.0121	1.49	0.58
VCAM1	TCGA-KIRP	0.0050	2.28	1.19
VCAM1	TCGA-LGG	0.0012	0.56	-0.84
VCAM1	TCGA-LUSC	0.0392	0.74	-0.44
VCAM1	TCGA-THCA	0.0187	0.32	-1.62

The clustering analysis separated CTC marker genes into two major groups: (*i*) *CD44, CXCR4, ICAM1, CCR1*, and *IL1R1* where high expression correlated with poor survival for low grade gliomas (LGG), kidney renal clear cell carcinoma (KIRC), for glioblastoma (GBM), kidney renal papillary cell carcinoma (KIRP) or lung squamous cell carcinoma (LUSC) (**Figure 3C**).

The second gene cluster (*ii*) includes *TGFBR2, IL6ST, IL6R, CDH1*, and *IGFBP5*. In this group we observed a correlation between high expression and better prognosis in KIRC and worse prognosis in KIRP and LGG.

As discussed in the previous sections, molecules included in the analysis promote CTC cluster formation or metastasis in functional studies. Indeed, the results of clustering analysis suggest that high expression of genes from the first cluster predicts a rapid disease progression in multiple cancers. Conversely, the second cluster contains more genes whose expression promotes cancer progression in a cancer specific manner.

However, in some cases focused investigations contradict prediction of patient survival based on the TCGA dataset (**Table 3**, upper right triangles in **Figure 3B** depict approximates for HR collected from the literature). Specifically, high expression of stem cell marker *CD44* corresponded to poor prognosis in kidney cancers (KIRC, KIRP) in TCGA data and, accordingly, high CD44 and b-catenin immunostaining correlated with advanced stage, although no significant correlation with survival could be observed in a specific focused study (117). However, other reports communicated a correlation between high CD44 levels and decrease of progression free survival in renal cell carcinoma after treatment with multi-targeted tyrosine kinase inhibitor (84). Consistent with the literature, high *CD44* expression predicts inferior prognosis in LGG and GBM TCGA cohorts (85, 86). The only case of association between *CD44* expression and positive thyroid cancer prognosis contradicts to the literature (87) (**Figure 3B**).

**Table 3:** Comparison between prediction of patient survival based on TCGA dataset and review of published literature.

Gene ID	Cancer type	Literature reported HR for patients with high level of gene or corresponding protein in the tumors.	TCGA calculated HR for patients with high expression of gene in the tumors	Reference
CD44	Renal cell carcinoma	high	high	(84)
CD44	Low grade glioma	high	high	(85, 86)
CD44	Thyroid	high	low	(87)
CXCR4	Breast	high	low	(88)
CXCR4	Thyroid (CD8 low)	high	low	(89)
CXCR4	Lung	high	low	(90)
ICAM1	Breast	low	low	(91)
ICAM1	Breast	high	low	(92)
CDH1	Colorectal	low	low	(93, 94)
CDH1	Low grade glioma	high	high	(95)
CDH1	Kidney renal papillary cell carcinoma	high	high	(96)
CDH1	Squamous cell carcinoma	low	low	(97)
IL6R	Glioblastoma	high	high	(98)
IL6R	Lung adenocarcinoma	low	low	(99, 100)
IL6R	Kidney renal clear cell carcinoma	high	low	(101, 102)
NOTCH1	Kidney renal clear cell carcinoma	high	low	(103, 104)
NOTCH1	Low grade glioma	high	low	(105, 106)
TGFBR2	Lung adenocarcinoma	low	low	(107, 108)
TGFBR2	Lung squamous cell carcinoma	low	high	(107, 108)
TGFBR2	Breast cancer	low	high	(109, 110)
TGFBR2	Breast cancer	high	high	(111)
EPCAM	Breast cancer	high	low	(112)
EPCAM	Low grade glioma	high	low	(113)
EPCAM	Kidney renal clear cell carcinoma	low	low	(114)
EPCAM	Kidney renal papillary cell carcinoma	low	low	(114)
VCAM1	Kidney renal clear cell carcinoma	low	low	(115, 116)

It was reported that high *CXCR4* expression corresponds to bad prognosis for breast (88), lung (90) and colorectal (118) cancers contradicting TCGA-based findings (**Figures 3B, C**). It was recently reported that in advanced CD8 negative thyroid cancer, high expression of CXCR4 and its ligand CXCL12 (SDF-1) correlates with bad prognosis, thus contradicting to TCGA data (89).

In contrast, *ICAM1* expression is associated with favorable prognosis in the breast cancer TCGA cohort, consistent with similar survival analysis of NCBI GEO dataset and repression of the lung metastasis in spontaneous breast cancer metastasis model (91) and contradicting another paper reporting pivotal role of the *ICAM1* in the CTC cluster formation, trans-endothelial migration and metastasis in breast cancer (92).

Thus, the positive associations between expression of CD44, CXCR4 and ICAM1 for thyroid, lung and breast cancers in TCGA dataset are not consistent with the literature suggesting that the first cluster is indeed represents genes whose high expression correlates with inferior prognosis consistent with their role in the CTCs biology.

Further we compared TCGA prediction with the literature for a few genes from the second cluster to address the question if they have more tissue specific roles in cancer metastasis.

One of such genes is CDH1 (E-cad protein) whose high expression was a predictor of better prognosis for colorectal cancer in agreement with TCGA data (93, 94). Again, consistent with TCGA data, high protein staining of E-cad in kidney renal papillary cell carcinoma was associated with worse prognosis, and no association was detected for kidney renal clear cell carcinoma (96). Recent analysis of E-cad in the cohort of NSCLC with 66% cases representing squamous cell carcinoma identifies E-cad as a positive prognostic factor consistent with TCGA data (97).

When astrocytomas, oligodendrogliomas and oligoastrocytomas were analyzed, the loss of E-cad immunostaining and hypermethylation of its promoter were associated with worse prognosis contradicting TCGA data, although, gene expression analysis was not performed (95). In contrast, consistent with TCGA data, a positive association between higher E-cad expression and worse prognosis was reported in the low-grade gliomas and in glioblastoma (119, 120).

IL6 receptors IL6ST and IL6R are involved in the CTC heterotypic interactions in breast (16) and colorectal cancers (31). Consistent with the literature, expression of IL6R has strong prognostic value in glioblastoma (98) and in lung adenocarcinoma (99, 100). In contrast, in kidney clear cell carcinoma we found a contradiction between the literature and TCGA data concerning the biomarker potential of IL6R expression: good predictor according to the literature (101, 102), and poor predictor according to TCGA data. Thus, the role of IL6R expression in cancer can be considered tissue specific.

The member of TGFb receptor family - TGFBR2 is a tumor suppressor in lung cancer, and the loss of TGFBR2 expression is associated with worse prognosis of both squamous cell cancer and adenocarcinoma (107, 108). Accordingly, TGFBR2 mutation predicts lung cancer resistance to checkpoint inhibitors (121). Thus, the literature supports prediction of TCGA dataset regarding the role of TGFBR2 in LUAD progression and contradicts association of high TGFBR2 with negative prognosis in LUSC. In breast cancer, reduced expression of TGFBR2 is associated with worse prognosis contradicting the TCGA data (109) especially in ER positive patients (110), while the report by Gao and coauthors is in line with the TCGA data (111). Theoretically, these contradictions might be connected with the presence of *TGFBR2* mutations which were not investigated in these published reports. Little is known about the influence of the TGFBR2 on glioma survival, however TCGA prediction of the negative association might be valuable since TGBFR2 compensates for inhibition of PDGFR, thereby promoting survival (122).

NOTCH1 activation as measured by the immunostaining against NOTCH intracellular domain correlates with poor prognosis of kidney renal clear cell carcinoma (KIRC) (123). In turn, high total *NOTCH1* immunostaining is associated with progression of kidney renal clear cell carcinoma contradicting TCGA prediction (103, 104). Likewise, in contrast to TCGA data, literature suggests association of NOTCH1 expression and glioma progression by modulating CXCL12/CXCR4 (105, 124).

In contrast, a recent meta-analysis revealed that *NOTCH1* expression does not correlate with overall survival in adenocarcinoma, although DLL4 and HES1 were associated with worse prognosis (125).

Measurements of *VCAM1* in KIRC revealed association of high expression with good prognosis consistent with TCGA data analysis (115, 116). In turn, for KIRP we found no published data that can validate the association of *VCAM1* high expression with good prognosis observed for the TCGA dataset.

*EPCAM* expression was associated with favorable prognosis of breast cancer in TCGA data, however immunohistochemical analysis has shown that it is associated with worse prognosis specifically in the basal-like and luminal B HER2+ subtypes (113). However, in the HER2+ subtype, *EPCAM* was also reported to be associated with worse prognosis (112). Again, in LGG the protein level of *EPCAM* was associated with poor prognosis, which contradicts to the TCGA trends (113). In thyroid cancers, the presence of EPCAM cleavage product was associated with more aggressive disease progression, although gene expression was not measured in this report (126). Finally, in agreement with the TCGA dataset, high EPCAM expression was associated with better prognosis in kidney cancers (114).

Overall, after comparison of TCGA data with the literature, it is possible to conclude that genes of the first cluster (top, **Figure 3B**) are mostly predictors of poor prognosis, whereas genes of the second cluster (bottom, **Figure 3B**) predict survival in a cancer type-specific manner (**Figure 3B**).

For interrogation of TCGA expression and survival data, we used standard analytic tools from the TCGA project portal GDC (127, 128). The discrepancies between results of TCGA data analysis and the literature could originate from different experimental methods used to assess gene expression, or different cohorts of patients and different treatment regimens among others. Thus, results of positive or negative gene association with patient survival require independent verification to identify or to confirm reliable biomarkers of disease progression and potential targets for drug development.

## 6 Molecules involved in the CTC heterotypic interaction and known drug targets

Analysis of the TCGA data and the literature revealed that high expression of molecules involved in the CTC heterotypic interactions predicts survival in many cancer types. Accordingly, as it is discussed in the previous sections, these molecules are pivotal for metastasis and therefore sometimes represent targets of clinically approved or experimental cancer drugs. Specifically, results of TCGA dataset analysis suggest poor prognosis for IL6 overexpressing low grade gliomas and glioblastomas. Indeed, pre-clinical data demonstrated that IL6 blockade combined with CD40 stimulation sensitized glioblastoma to immune checkpoint inhibitors and improved survival (129, 130). Likewise, pre-clinical investigations revealed that targeting of the IL6 signaling might be beneficial for other cancers as well, where bad prognosis is associated with high IL6 level such as renal cell carcinoma (131, 132), non-small cell lung cancer (133), and breast cancer (134). We found a single, currently suspended clinical trial of the IL6R antibody tocilizumab for gliomas and glioblastoma treatment (NCT04729959), trials for metastatic breast cancer (NCT03135171), non-small lung cancer among others (NCT04940299, **Table 4**). Targeting of IL-6 improves immunotherapy outcome in mice models (155, 156). However, IL6-specific antibody siltuximab demonstrated no efficiency against renal cell carcinoma (157) and prostate cancer (158).

**Table 4:** Potential off-label applications of drugs targeting molecules involved in the heterotypic CTCs interactions.

CTC cancer type	Target	Target ligand(s)	Drug	Current therapeutic applications	Potential therapeutic applications	References and clinical trials
Breast, Colorectal	IL6ST IL6R	IL6	Siltuximab, tocilizumab	Castleman disease, Rheumatoid arthritis, GBM, LGG, LUSC, BRCA		(129, 130, 133–135) NCT04729959 NCT04940299 NCT03135171
Breast	IL1R1	IL1	anakinra	rheumatoid arthritus, MM, BRCA, colorectal cancer	GBM, LGG, KIRP, KIRC LUSC	(136–141) NCT00635154 NCT01802970 NCT02090101
Breast	IL1R1	IL1	isoanakinra	Solid cancers	GBM, LGG, KIRP, KIRC LUSC	NCT04121442 NCT00072111
Breast	CD44	Hyaluronic acid	RG7356	NA	Solid cancers, AML, GBM	(142–145)
Breast	CXCR4	CXCL12	Ulocuplumab	Multiple myeloma	THCA	(146)
Breast	CXCR4	CXCL12	AMD3100/Plerixafor X4P-001	hematopoietic stem cell (HSC) mobilizer, colorectal cancer, glioblastoma	THCA	(147)
Breast	CXCR4	CXCL12	X4P-001	Triple negative Breast cancer	THCA	NCT05103917
Breast	CXCR4	CXCL12	MB1707	Advanced cancers, NSCLC, breast cancer	THCA	NCT05465590
Breast	TGFBR2	TGFB1	Vactosertib	Solid cancers	LGG, LUSC, BRCA	(148, 149)
Hepatocellular	EPCAM	CAMs	catumaxomab (anti-EpCAM x anti-CD3), bladder, ovarian cancers	malignant ascites	GBM	(150–153)
Lung	ICAM1	ITGAM	Lifitegrast LFA-1/ICAM-1 antagonists	dry eye disease	GBM, LGG, KIRP, KIRC	(153, 154)

MM, multiple myeloma; AML, acute myeloid leukemia.

IL1R1 expression predicts poor survival in nearly the same set of cancer types as IL6R. There are multiple clinical trials testing IL1R agonist an anti-rheumatoid arthritis drug anakinra against multiple myeloma (136), metastatic breast cancer, and colorectal cancer (159), listed in **Table 4**. However, we didn’t find any specific records for gliomas, lung or kidney cancers. Still, several preclinical investigations have shown that targeting of IL1 signaling in GBM (137, 138), LGG (139) kidney (140) and lung cancer (141) suggest its potential clinical usefulness.

Expression of adhesion molecule ICAM1 also predicts poor prognosis for several cancer types, closely mimicking the effects observed for the *IL1R1* and *IL6R* genes. Specifically, low ICAM1 expression corresponds to better survival in GBM. Indeed, bispecific CAR-T cells against EPCAM and ICAM1 elicited good response in GBM mice model (153), consistent with other preclinical studies (160, 161). Similarly, CAR-T cells targeted against ICAM1 were successfully tested in mice models of gastric (162), thyroid (163, 164), and triple negative breast cancer (165).

ICAM-1 conjugated with a cytotoxic drug was extensively tested for multiple myeloma (166) and another bispecific anti-CD38-ICAM-1 drug for multiple myeloma is under development (167). The vaccine targeting ICAM-1 is also at the early stage of clinical investigation against ICAM-1 overexpressing bladder cancers (168) or lung cancer (NCT02043665). However, so far, we did not find reports on ICAM-1 targeted therapies clinically tested against gliomas and kidney cancers.

A stem cell marker CD44 predicts poor prognosis in renal cancers and in gliomas. The CD44-specific antibody RG7356 in clinical trials showed moderate efficiency in solid tumors (142) and in acute myeloid leukemia (169). There is also multiple evidence suggesting potential efficiency of CD44 targeting for the treatment of GBM, although additional clinical validation is clearly needed (143–145).

Catumaxomab (genetically engineered bivalent anti-EPCAM and anti-CD3 antibody) is approved for the treatment of malignant ascites (150) and it has been also used experimentally for the treatment of bladder (151) and ovarian (152) cancers. Bispecific CAR-T simultaneously targeting EPCAM and ICAM-1 demonstrated promising results in mice models of gastric and pancreatic cancers (153).

Anti-CXCR4 antibody demonstrated efficiency in multiple myeloma in combination with lenalidomide or bortezomib plus dexamethasone (146), and several related clinical trials are ongoing. A CXCR4 inhibitor AMD3100/Plerixafor was approved by FDA as a hematopoietic stem cell mobilizer and it was recently tested in humans against pancreatic and colorectal cancers as the potential inducer of the immune response (147). Also, preclinical studies showed that inhibition of the CXCR4 might be potentially efficient against other cancers including GBM (170, 171), and the first human clinical trial of plerixafor as an adjunct to combined chemoradiotherapy was conducted in newly diagnosed GBM patients (172) achieving median overall survival of ~21 months. This is a significant improvement over ~17 months period characteristic for the standard chemoradiotherapy (173).

Finally, gamma secretase inhibitors showed therapeutic effects only in CNS tumors and desmoids (174). Targeting of TGF-β receptor is also in development and in clinical trials (148). In turn, anti-VCAM antibodies dramatically reduced pancreatic ductal adenocarcinoma progression in mice models (175, 176).

In **Table 4**, we summarized drugs targeting molecules involved in the CTC heterotypic interactions.

## 7 Conclusions

Analysis of the literature describing factors leading to formation of CTC clusters revealed three major features. *First* - the presence of either heterotypic or homotypic CTC aggregates often means unfavorable prognosis and predicts metastasis in many cancer types. Targeting the formation of such clusters is a valuable strategy for metastasis suppression (4, 6, 17). *Second* - cells carry mesenchymal (Vimentin) and epithelial (E-cad) markers together, which is a hallmark of intermediate epithelial associated with stemness of cancer cells (177, 178). *Third* - in turn, intermediate Mesenchymal- Epithelial state frequently coincides with polyploidy as it was shown in lung and colorectal cancers (179, 180). In lung cancer, polyploidy was accompanied by the interaction with WBCs, which were identified as neutrophils or PMN-MDSCs.

It is well established that both polyploidy/mixed EMT phenotype and immunosuppressive PMN-MDSC and TAM contribute to cancer progression, however, how the interaction between them mediates metastatic advantage is yet to be investigated.

Taken together, these findings highlight common mechanisms of metastasis with implication for drug development and cancer treatment.

## Author contributions

JMR wrote the text, edited figures and performed data analysis, TM wrote the text and prepared figures, AAB conceptualization and text writing and editing, JMR, AAB and TM These authors contributed equally to this work and share first authorship. OAR, DAD, VVP, and IVA collected related literature, compiled data and wrote the manuscript. All authors contributed to the article and approved the submitted version.
